# Modeling traumatic brain and neural injuries: insights from zebrafish

**DOI:** 10.3389/fnmol.2025.1552885

**Published:** 2025-03-27

**Authors:** Lada Murashova, Vyacheslav Dyachuk

**Affiliations:** Almazov National Medical Research Centre, Saint Petersburg, Russia

**Keywords:** zebrafish, neuroregeneration, traumatic neural injury model, posttraumatic cellular interactions, post-traumatic neurogenesis

## Abstract

A traumatic injury to the nervous system has significant consequences for mammals, including long-term disability, loss of functions, and neuropathic pain. In contrast to mammals, zebrafish (*Danio rerio*) exhibits a markedly enhanced neuroregenerative capacity, which can be attributed to the phenomenon of adult neurogenesis and to the distinctive characteristics of the inflammatory response at the injury site. The post-traumatic recovery of zebrafish under different experimental injury conditions was demonstrated in numerous studies, which has substantially advanced our understanding of the cellular and molecular mechanisms of neuroregeneration in this animal. In view of the significant differences in molecular mechanisms depending on the injury site, lesion severity, and harmful agents, selecting an appropriate model for investigations is of paramount importance. This review discusses some approaches to modeling neural injury in zebrafish and considers the effect of cellular interactions in post-traumatic neurogenesis, with focus on the animal’s age and the specific damaging factor that may be used to select an optimum model for certain nervous system lesions.

## Introduction

In recent years, the zebrafish (*Danio rerio*) has become a promising model for neuroscience research, both in normal conditions and in disease modeling. The number of articles on this topic has dramatically increased over the past decade.

Eggs, early embryos, and larvae are optically transparent. This transparency allows detection of fluorescent proteins, in particular in cells of transgenic fish (Choe et al., 2021), e.g., the development and migration of Schwann cells (Cunningham and Monk, 2018). In post-traumatic, Tau-linked conditions in Tau-GFP reporter zebrafish (Alyenbaawi et al., 2021), the vital fluorescent dyes can be used as makers in early developmental stages to analyze behavior of single cells (Cunningham and Monk, 2018; Bensimon-Brito et al., 2016). Transparent strains of adult fish (Casper and Crystal) are available for *in vivo* imaging in various research fields (Antinucci and Hindges, 2016).

From a morphological viewpoint, zebrafish is a valuable model across vertebrates due to its conserved nervous system parts. Both zebrafish and mammals possess a tripartite brain structure consisting of a forebrain, a midbrain, and a hindbrain (Saleem and Kannan, 2018). A similarity of gene expression patterns, neurotransmitters, receptors, and regional connectivity within well-defined substructures exists between fish and mammals (Kozol et al., 2016). Moreover, zebrafish exhibits fast neural development, with major central nervous system (CNS) regions being recognizable at approximately 24 hpf and most cell type circuits and nuclei being functional by 3 days post-fertilization (dpf) and continuing to grow and differentiate thereafter (Kaslin and Ganz, 2020). It was also shown that the developmental changes of gene expression patterns in the brain of a 40–59-yr-old human and that of a 1–2-yr-old zebrafish are somewhat similar (Nakajima et al., 2021). It is noteworthy that the cognitive function also declines with age in zebrafish, a phenomenon that is attributed to subtle changes in cellular and synaptic integrity (Adams and Kafaligonul, 2018).

Zebrafish is a valuable model for regeneration studies due to its high regenerative capacity. This animal can successfully regenerate fin, heart, pancreas, liver, kidney, skin, hair cells of lateral line, and CNS (Marques et al., 2019). A differences between zebrafish and mammals is the presence of multiple neurogenic niches in the CNS even in adult individuals (Zupanc and Sîrbulescu, 2011; Kizil et al., 2012; Schmidt et al., 2013). The zones of constitutive neurogenesis that exist in the zebrafish’s CNS throughout life are located in different parts of the brain, in contrast to rodents whose proliferative activity in the brain is found in two major areas (Diotel et al., 2020). Previous studies showed that a spinal cord injury can effectively regenerate in larvae within approximately 48 h (Ohnmacht et al., 2016), while it takes 4–8 weeks for adult zebrafish to regenerate motor neurons at the injury site (Reimer et al., 2008). Moreover, zebrafish can regenerate various neural tissues including the retina (Sherpa et al., 2008), optic nerves (Harvey et al., 2023), vagal nerves (Isabella et al., 2021), other peripheral nerves (Ceci et al., 2014; Bremer et al., 2017), and the brain (Zupanc, 2006; Kishimoto et al., 2012; Pose-Méndez et al., 2023). Following a damage to neural structures in zebrafish, certain cell populations provide rapid proliferation and replacement of dead cells. These include radial glia cells (Kroehne et al., 2011), neurogenic progenitor cells (Kizil et al., 2012), and oligodendrocyte precursor cells (Tsata et al., 2020). In the retina, Müller glia have been shown to undergo reprogrammed to regenerate retinal neurons (Lahne et al., 2020). Contrary to observations on mammals, zebrafish does not exhibit extensive glial scarring following CNS injury. The lack of scarring creates a permissive environment favorable for axonal regrowth and tissue repair (Mokalled et al., 2016). The cellular and molecular mechanisms of this process can be translated to mammals. Therefore, the use of zebrafish as a model organism for nervous system research is highly promising and provides many new opportunities for extending our knowledge of neural regeneration and for disease modeling.

Zebrafish is suitable for research purposes at all ages, with larvae and sexually mature animals (6–8 months post-fertilization) being the most commonly used in neuroregeneration studies. However, depending on the specific objectives of the experiment, juvenile and older animals can also be selected. The transparency of larvae facilitates visualization at the cellular level, enabling observation of glial cell and neuronal behavior in transgenic reporter lines, assessment of neuronal excitation (calcium imaging), and analysis of axonogenesis (Hecker et al., 2020) and synaptogenesis in a living organism. Additionally, it allows rapid screening of pharmacological substances and their effect on labelled cells. Despite the possibility to study fundamental behavioral responses, the behavioral repertoire in larvae is, however, not as extensive as that in adults.

Adult zebrafish possesses the fully developed immune and endocrine systems, thus, providing the opportunity to elucidate neuroimmune interactions and the effect of sex on neuroregeneration. Furthermore, the phases of regeneration can be more clearly distinguished in adult animals, and, due to their more complex behavior compared to larvae, the functional recovery or lack thereof of memory, learning ability, and social behavior can also be assessed. Adult zebrafish suits well for studying the ageing processes and their effect on the regenerative response, which can provide valuable clues for translational research.

### Approaches to traumatic injury modeling

**Figure 1:**
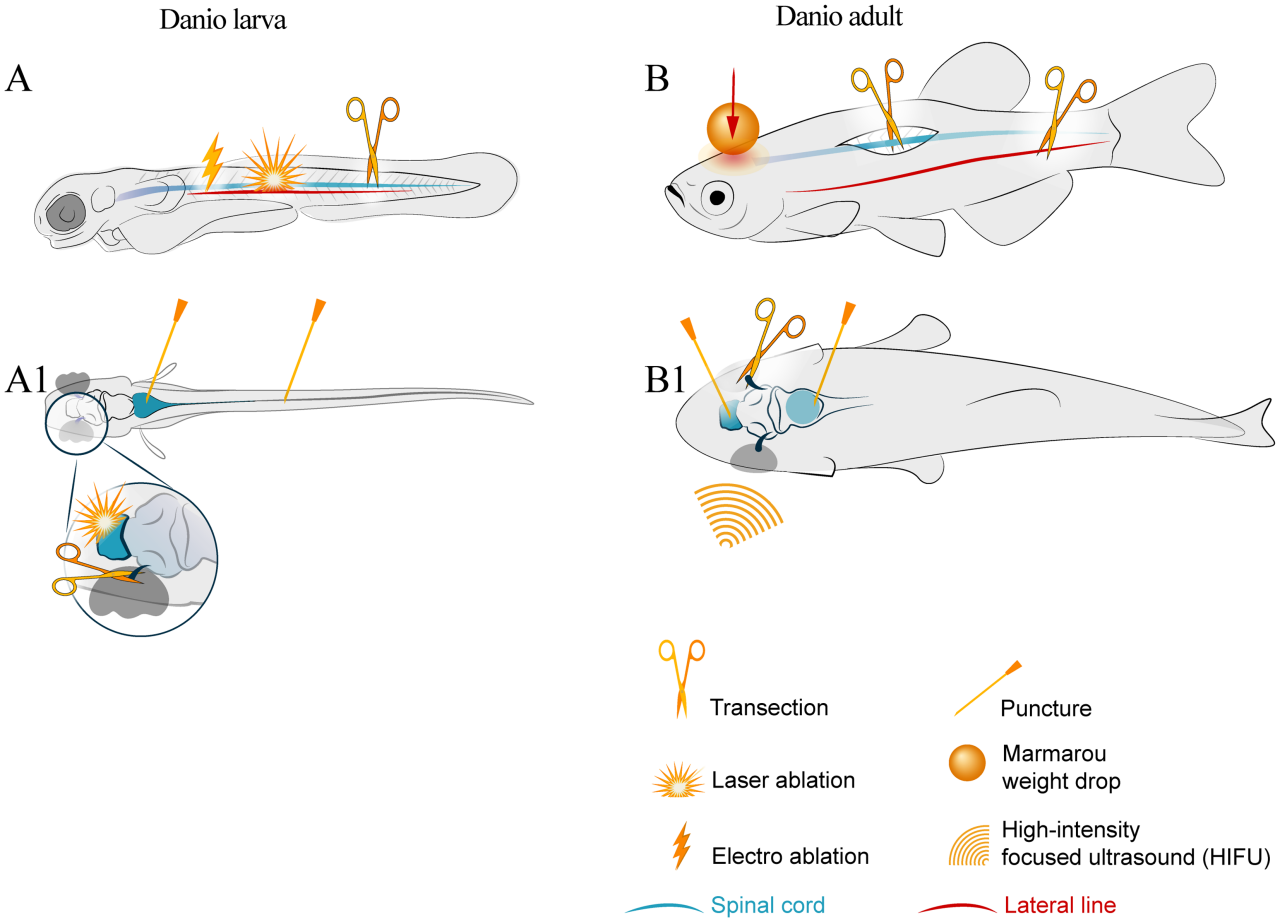
Schematic of injury sites in larval and adult zebrafish. The notation keys of injury types are presented in the bottom right corner. **(A)** larval lateral view; symbols show laser ablation of the cells in the central nervous system, electro-ablation of the lateral line axons, transection of the spinal cord by the edged tool. **(A1)** larval dorsal view with symbols indicating brain stem and spinal cord puncture trauma and optic nerve transection. **(B)** adult lateral view and **(B1)** adult dorsal view with symbols of spinal cord and optic nerve transection, lateral line axotomy, stab wound of the brain areas, and whole brain trauma by Marmarou weight drop and high intensity ultrasound.

In this review, we discuss traumatic injury modeling, both local and systemic, in zebrafish. The methods for chemical and genetic ablation of certain cell populations and stroke modeling are too extensive to fit the scope of this review. The most common approaches to modeling neural injuries are shown in Figure 1.

The closed head trauma model in zebrafish is inflicted in different ways. It closely mimics a clinical situation with loss of consciousness, seizures, brain edema, and hemorrhage. With variations in impact force, it is possible to obtain scalable models of closed head trauma, from slight concussion to severe brain damage. In some investigations, it was caused in adult wild-type AB zebrafish using pulsed high intensity focused ultrasound (pHIFU). Two modes of pHIFU were set up, behavior tests were performed (Novel Tank test and Shoaling Test), and protein expression changes in the brain tissue were assessed. The post-injury changes were shown to be dose-dependent (McCutcheon et al., 2017).

Another method for traumatic brain injury in adult zebrafish is based on head injury with dropped weight. This model was designed for rodents (Marmarou et al., 1994).

This approach was applied to create a reproducible cost-effective traumatic brain injury model in adult zebrafish and analyze the gene expression and signaling pathways involved in post-traumatic recovery (Maheras et al., 2018).

Mild modifications were made to circumvent skull damage in the experimental animals. For this, adult wild-type AB, *Tg[nestin:GFP]*, *Tg[gfap:EGFP]* transgenic lines, and *albino* and *roy; mitfa* mutant lines of zebrafish were used. In that study, behavior tests were performed, gross pathology examination carried out, cell apoptosis and proliferation examined, and the Sonic hedgehog pathway in post-traumatic regeneration in the cerebellum demonstrated (Hentig et al., 2021b).

These methods are not suitable for larvae due to their small size. Nevertheless, some approaches were made for close head trauma modeling in early developmental stages of zebrafish. One of them was used to detect abnormal Tau protein in *Tg[Tau4R-GFP]* larvae after traumatic brain injury (TBI). In that study, the post-traumatic hemorrhage in *Tg(gata1a:DsRed)* larvae was assessed, and bursts of neuronal excitation during TBI causing seizures in *Tg(elavl3:CaMPARI)^ua3144^* larvae were documented. The method can be described as follows: zebrafish larvae (in their typical liquid growth medium) were loaded into a syringe with a closed valve stopper. A hit was applied on the plunger to produce a pressure wave comparable to shock waves experienced in the case of human blast injury. The study showed a correlation between the seizure intensity and the accumulation of abnormal Tau protein aggregates in the post-traumatic period (Alyenbaawi et al., 2021).

Another approach to closed head trauma modeling in larvae is the “concussion machine”: a cylindrical capsule filled with the E3 medium, which moves fast and stops abruptly (Beppi et al., 2022). The TBI caused by this method is milder than that in the above-described case. Nevertheless, behavioral changes were observed in that study.

For local open head trauma modeling, a stab wound is typically inflicted. A fine, sterile needle or glass capillary is inserted into a certain brain region of anesthetized fish: the telencephalon (Kishimoto et al., 2012; Kim et al., 2020; Lübke et al., 2022), optic tectum (Gan et al., 2020; Ueda et al., 2018; Shimizu et al., 2018), cerebellum (Wu et al., 2014), or hindbrain (Hannah et al., 2012). Local injury is caused to study cell apoptosis (Anand et al., 2021), proliferation (Kishimoto et al., 2012; Baumgart et al., 2012; März et al., 2011), signaling pathways (Kishimoto et al., 2012; Shimizu et al., 2018; Wu et al., 2014), and gene expression in the damaged zone (Kizil et al., 2012; Anand et al., 2021; Yin et al., 2018).

Stab wound has been sufficiently well developed for investigating post-traumatic neurogenesis and cellular crosstalk in the mature brain (März et al., 2011; Schmidt et al., 2014). While this technique is commonly applied to adult zebrafish, it has also demonstrated reproducible results on fish larvae (Gan et al., 2020). Study of post-traumatic neuroregeneration in zebrafish is particularly intriguing due to the age-related changes that occur on tissue, cellular, and subcellular levels and can begin 1 year post-fertilization (Van Houcke et al., 2021).

The spinal cord and peripheral nerves can be considered as linear structures. Thus, transection with scissors and crushing with jeweler forceps can be made to induce traumatic injuries in these regions, as described in Supplementary Table 1. Since the spinal cord is a component of the CNS, and nerves are part of the peripheral nervous system (PNS), these structures exhibit significant differences in cell composition and regeneration processes.

Spinal cord injury modeling protocols have actively been applied to both adult and larval zebrafish over the past 25 years. In case of adult animals, the typical transection localizations are at the level of brainstem/spinal cord transition zone (Becker et al., 1998), 3.5–4.0 mm caudally of the brainstem/spinal cord transition zone at the level of the 8th–9th vertebrae (Becker et al., 1998; Vajn et al., 2014), and at the level of dorsal fin, which corresponds to the 15th–16th vertebrae (Hui et al., 2014). For motor neuron activity research, traumatic injury can be applied at the level of the 24th vertebra (Stil and Drapeau, 2016). In larval zebrafish, the spinal cord can be transected with a needle (Ohnmacht et al., 2016; John et al., 2022; Hossainian et al., 2022) or a “glass scalpel” made from broken glass capillary (Briona and Dorsky, 2014).

Extensive research has been conducted on the spinal cord regeneration in zebrafish, and the regeneration process studied in sufficient detail.

To elucidate the axonal regeneration in the PNS, studies focused on large visible nerves: the optic nerve, posterior lateral line, and vagal nerve (Harvey et al., 2023; Isabella et al., 2021; Graciarena et al., 2014). Axotomy protocol was also designed for laser ablation of trigeminal nerve axons in larvae (O’Brien et al., 2009), single dorsal root ganglion axons (Nichols and Smith, 2020), and for motor nerve transection (Rosenberg et al., 2012). These studies also considered neuron–glia interactions (Ceci et al., 2014; Graciarena et al., 2014) and metabolomic changes in optic nerve regeneration (Meehan et al., 2023). Various methods were employed to model spinal cord injury, including transection using a needle in larval zebrafish (Harvey et al., 2023), transection using a glass capillary in adult and aged zebrafish (Graciarena et al., 2014), nerve crush using fine forceps (Meehan et al., 2023; Diekmann et al., 2015), laser axotomy (Isabella et al., 2021), and electroablation (Ceci et al., 2014; Moya-Díaz et al., 2014).

In the case of nerve crush method, a mechanical force is applied to a nerve without severing it completely, thus, preserving its complete structural integrity. This method ensures continuity of the surrounding connective tissue, thereby creating a favorable environment for enhanced regeneration. In the case of nerve transection, the nerve is completely severed, resulting in a more profound damage. When both axons and connective tissues are severed, this typically leads to a more complex healing process.

Electroablation represents a noteworthy approach to focal cell ablation and axotomy. The use of microelectrodes allows ablation of single lateral-line mechanosensory neuromasts and posterior lateral-line axons in larval and adult zebrafish, thereby providing the opportunity to observe neuroregeneration in damaged tissues (Moya-Díaz et al., 2014). Two modes of electroablation can be applied: radiofrequency (thermal protein coagulation in damaged tissue) (Widmann, 2009) and irreversible electroporation of membranes of damaged cells without thermal damage to tissue (Thomson et al., 2015), which is typically used for treatment of large animals and humans. It is relevant to note that the use of electroablation in zebrafish for traumatic injury research is still a relatively novel technique, which requires further investigation to fully understand its potential and limitations. Nonetheless, the opportunity to induce precise and focal injuries in zebrafish using electroablation provides a valuable tool for studying the cellular and molecular mechanisms underlying the post-traumatic neuroregeneration in the CNS and PNS.

Laser ablation is another noninvasive technique for neural damage that has been applied to zebrafish larvae (Liu and Fetcho, 1999; Roeser and Baier, 2003; Muto and Kawakami, 2018) due to their optical transparency and the clear visibility of structures to be ablated. However, this technique can also be used for adult fish (Tikhonova et al., 2022). For precise laser ablation using the two-photon excitation microscopy, cells to be ablated should be distinguished based on their optical characteristics. This requires distinct cell labeling with vital stains (Liu and Fetcho, 1999) or fluorescent protein expression in transgenic zebrafish (Roeser and Baier, 2003; Muto and Kawakami, 2018; Hiyoshi et al., 2021; Xiao et al., 2022). This method is useful for identifying the functions of specific cell populations in a particular body area or even single cells. For adult fish, a diode laser can be used, which, with proper tuning of focal distance and laser power, induces focal injuries, with above-located tissues remaining intact (Tikhonova et al., 2022). However, this approach has some disadvantages, including the high cost of equipment required and the time-consuming procedure in case where many cells are to be ablated.

### Retinal injury modeling

Zebrafish has been found as an exceptionally useful model for studying retinal injury and subsequent regeneration due to its remarkable capacity to regenerate retinal neurons after damage. The following approaches can be used to investigate retinal regeneration: laser-induced injury (DiCicco et al., 2014), chemical lesions, e.g., intravitreal injections of ouabain (Barrett et al., 2022), physical damage by poking with a needle to make a stub wound injury (Sharma and Ramachandran, 2019) or stab wound technique (Senut et al., 2004), thermal injury (Bernardos et al., 2005), or combined approaches. A range of mechanical models of retinal injury in zebrafish have been shown to offer valuable clues into the mechanisms of retinal regeneration. However, these models have distinct advantages and disadvantages. Needle poke and stab wound injuries, e.g., while creating uniform and localized lesions, can give also variable results due to individual differences in reaction. In contrast, laser-induced injuries offer high precision but necessitate specialized equipment, and heat-induced injuries permit controlled severity, while, however, causing non-specific damages. It is, therefore, essential to take these advantages and disadvantages into account when selecting the most appropriate model for addressing specific research issues related to retinal regeneration.

### Summary of approaches

To summarize the overview of approaches to modelling nervous system injuries, the following conclusions can be drawn. The models of closed head injury in adult fish are the closest to the situations observed in clinical practice. The key advantage of the pHIFU method consists in non-invasive and scalable injuries caused. This allows researchers to model a wide spectrum of TBI severities without causing mechanical skull damage. However, it requires specialized equipment and expertise, which potentially limits availability. Conversely, the dropped weight method offers simplicity and cost-effectiveness, facilitating reproducible results without the need for advanced equipment. However, it is less precise than pHIFU, as controlling the force and localization of the injury is more challenging. Both methods have contributed substantially to understanding TBI in zebrafish. The pHIFU model is particularly suited for studies requiring precise control over injury severity and localization such as dose–response analyses of molecular pathways. Conversely, the simplicity of the latter makes it a perfect choice for large-scale studies investigating into genetic and cellular mechanisms.

Whilst it is not yet technically feasible to model isolated closed head injuries in zebrafish larvae, it is possible to induce shock wave-induced injury that involves the entire body. The above-described approaches also allow potential assessment of the embryonic nervous system injury and the consequences for body development. The transparency of zebrafish larvae makes it possible to detect cellular damage, hemorrhages, and accumulation of aggregates of abnormal proteins. Furthermore, the use of transgenic reporter lines such as *Tg(plvap:EGFP)* (Umans et al., 2017) gives an opportunity for detection of alterations in the brain microcirculation and the blood–brain barrier and their subsequent effect on the animal’s development.

Stab wound, electroablation, and laser ablation are the techniques that permit the evaluation of the specificity of the response to injury in specific regions of the nervous system. However, the translational potential of such interventions is limited. While stab wound provides the simulation of open brain injury in a specific brain region with subsequent inflammation of the tissues along the wound channel, electro- and laser ablation are more suitable for the study of compensatory mechanisms after the loss of parts of the nervous system or specific neurons, for elucidating the formation of new neuronal pathways, behavioral changes, and for identification of new interactions between specific parts of the nervous system and other systems of the body. Furthermore, stab wound is an appropriate model to study the cellular and molecular features of nervous system regeneration in a particular area of the nervous system in animals of different ages. This may be important in the further development of therapeutic strategies for mammals.

Although surgical spinal cord transection has not provided simulation of the clinical scenario in humans, this experimental model remains a common classic technique employed in both zebrafish (Becker et al., 1998) and rodent models (Kjell and Olson, 2016). This approach has yielded substantial data on the characteristics of spinal cord regeneration in zebrafish, thus, facilitating the development of therapeutic strategies for mammals, including cell therapy and utilization of the neurotrophic factors. Further studies on this model may clarify the influence of epigenetic regulation on spinal cord regeneration in animals of different ages and the influence of pharmacological agents on regeneration.

It is evident that both nerve crush and transection injuries, modelled in zebrafish, can give rise to divergent regenerative outcomes and mechanisms. Nerve crush has been shown to result in faster and more effective regeneration due to the preservation of supportive structures. Conversely, nerve transection has been demonstrated to pose greater challenges for recovery due to complete disruption of the nerve architecture. Understanding these differences is crucial for designing effective therapeutic strategies for nerve injuries in zebrafish models and for potential applications in humans.

### Cellular events in response to damage

The organism’s response to a nervous system injury is a multifaceted process involving diverse cell populations including neurons, radial glia, oligodendrocytes, microglia, Schwann cells in the PNS, fibroblasts, pericytes, blood cells (in particular neutrophils and macrophages), and mast cells. Such an intricate response necessitates coordinated interactions of multiple cell types.

#### Cellular dynamics

In one of the studies, zebrafish larvae at 3 dpf exhibited a remarkable capacity for motor function recovery within 48 h following spinal cord transection. Their individual motor neuron axons commenced entering the injury site as early as 12 h post-transection, with a substantial presence of regenerated axons observed by the 36-h mark (Ohnmacht et al., 2016). An extended period of proliferative activity was recorded at 5 dpf. Nonetheless, by 9 days post-injury (dpi), detectable neuronal presence was already observed within the damaged area (Anguita-Salinas et al., 2019). The results of studies with spinal cord electroablation in zebrafish larvae are generally consistent with those with mechanical transection of this structure, although there are differences in the timing of the inflammatory response between these experimental techniques (Anguita-Salinas et al., 2019).

In the adult zebrafish model, an immediate inflammatory response to spinal cord injury is manifested within hours post-injury, and the proliferative activity becomes apparent at 3 dpi (Hui et al., 2014). The spinal cord tissue remodeling is observed for three to 4 weeks, ultimately culminating in the functional recovery around the six-week mark (Becker et al., 2005). Despite this recovery of functional integrity within 42 dpi, up to 50% of axonal tracts located rostrally of the injury site remain unrepaired, and the deficiency of axonal myelination suggests incomplete regeneration. Of particular note is that even at 1 year post-injury, the spinal cord tissue in adult zebrafish cannot fully regenerate its anatomical integrity and myelination to the pre-injury level (Tsata et al., 2020).

Following damage, microglia actively aggregate at the injury site in adult zebrafish. The increase in cell number is related to migration and proliferation processes (März et al., 2011). In the context of spinal cord injury in zebrafish larvae, microglia are activated within the injury zone and are involved in phagocytosis of cellular debris (Morsch et al., 2015). Moreover, due to the disruption of the blood–brain barrier, bone marrow-derived macrophages also aggregate in the injury zone (Hui et al., 2010), with the primary function being to clear cellular debris and modulate the local inflammatory response.

A distinct population of stress-resistant cells is present along the wound edges in the case of spinal cord injury in zebrafish larvae. These cells can be categorized into subtypes associated with radial glia and neuronal progenitor cells. They are capable of differentiating into neurons that bridge the wound edges during the regeneration process. Furthermore, cells located on the rostral side of the lesion exhibit caveolin-1 expression, which augments the regenerative response and fosters axon growth in that region (Zeng et al., 2021).

Glial cells have been identified as the most actively proliferating cell population in the spinal cord injury model (Goldshmit et al., 2012). Member A of the acidic nuclear phosphoprotein 32 (*ANP32a*) enhance the motor neuron and ependymo-radial glia cell proliferation following post-spinal cord injury in zebrafish larvae (Lee et al., 2022). Significant radial glia proliferation and subsequent neurogenesis in the spinal cord of zebrafish larvae has also been noted (Briona et al., 2015).

After spinal cord injury in adult zebrafish, the number of oligodendrocyte bodies and myelin sheaths in the lesion site becomes reduced. Apoptotic cell death can be detected as early as 4 h post-injury (hpi). Oligodendrocyte progenitor cells in the spinal cord exhibit dynamic changes in morphology within the first 3 dpi, including the increase in size, the formation of long branching outgrowths, and the increased proliferative activity in response to experimental injury (Tsata et al., 2020). It has also been found that *olig2*-positive oligodendrocyte precursors, which do not express myelin basic protein (MBP), aggregate in the stab wound area in the damaged telencephalon hemisphere. Although their proliferation is not very active, it suggests migration to the injury site, albeit temporarily, because this phenomenon is not observed after 35 days (März et al., 2011).

The expression of *olig2* in the ventromedial region of the spinal cord in adult zebrafish significantly increases at 2 weeks post-injury, and these *olig2*-positive cells subsequently differentiate into *hb9*+ motor neurons (Reimer et al., 2009). After a stab wound to the telencephalon hemisphere, an increase in *mdka* expression is observed in quiescent radial glia cells of the damaged hemisphere in response to the injury, thus, indicating its role in the regulation of stem cell behavior (Lübke et al., 2022).

Such a stab wound also causes a slight decrease in GFAP expression in radial glia cell processes at 1 dpi, followed by a gradual increase from 3 to 14 dpi. The number of S100B-positive cells (radial glia cell bodies) also gradually increases from 8 dpi. GFAP-positive fibers in the injured hemisphere form hypertrophic swellings resembling cell bodies without nuclei, similar in appearance. Active proliferation of cells, including radial glia, is observed in the ventricular region of the damaged hemisphere (März et al., 2011). The radial glia’s ability to divide decreases with the animal’s age even in post-traumatic conditions. However, the formation of neuroblasts and subsequent neurogenesis appear to remain largely unaffected (Edelmann et al., 2013).

In zebrafish larvae, if an injury is inflicted to the spinal cord at 5 dpf, V2a interneuron bodies can be observed at the injury site as early as 9 dpi. These neurons exhibit the ability to receive signals from motor neurons and generate action potentials; however, they display distinct morphological and functional characteristics compared to mature V2a neurons. The persistence of these differences throughout the animal’s lifespan after the injury remains unclear (Vasudevan et al., 2021).

Regeneration of Mauthner cell axons post-laser ablation depends on the distance from the soma where the axon is severed, which is possibly regulated by regeneration-associated genes (RAGs). Although previous studies suggested incomplete functional recovery of these neurons post-injury, a successful regenerative response was observed with cAMP treatment following laser ablation in zebrafish larvae (Hecker et al., 2020).

A study highlighted the critical role of *pdgfrb +* cells in the structural repair of axons post-spinal cord injury in zebrafish larvae and adults. A marked expansion of the *pdgfrb +* cell population occurs around 7 dpi, coinciding in time with the initiation of axonal recovery after the injury (Tsata et al., 2021).

In adult zebrafish, the expression of *hb-egfa* significantly increases within the first 2 weeks post-spinal cord injury. Impairment of this factor was shown to negatively affect the axonal bridging between the injury edges and the post-traumatic neurogenesis (Cigliola et al., 2023).

After cerebellar injury in adult zebrafish, regeneration is primarily driven by neuroepithelial cells in the dorsal part of the ventricular zone, with radial glia cells playing a secondary role. Since the latter cells respond to injury, they are unable to be fully recruited to the lost cell population in the adult cerebellum. In juvenile fish, cerebellar regeneration is complete, leading to the regeneration of all damaged cell types (Kaslin et al., 2017).

A model of blunt-force head trauma in adult fish induces diffuse changes in the brain, with apoptosis-type neuronal death occurring predominantly in regions with high neuronal density. Constitutive neurogenic niches exhibit enhanced proliferative activity in response to the blunt force trauma, correlating with its severity. The proliferative response across the neuroaxis increases in a severity-dependent manner (Hentig et al., 2021a).

#### Major signaling pathways

**Figure 2:**
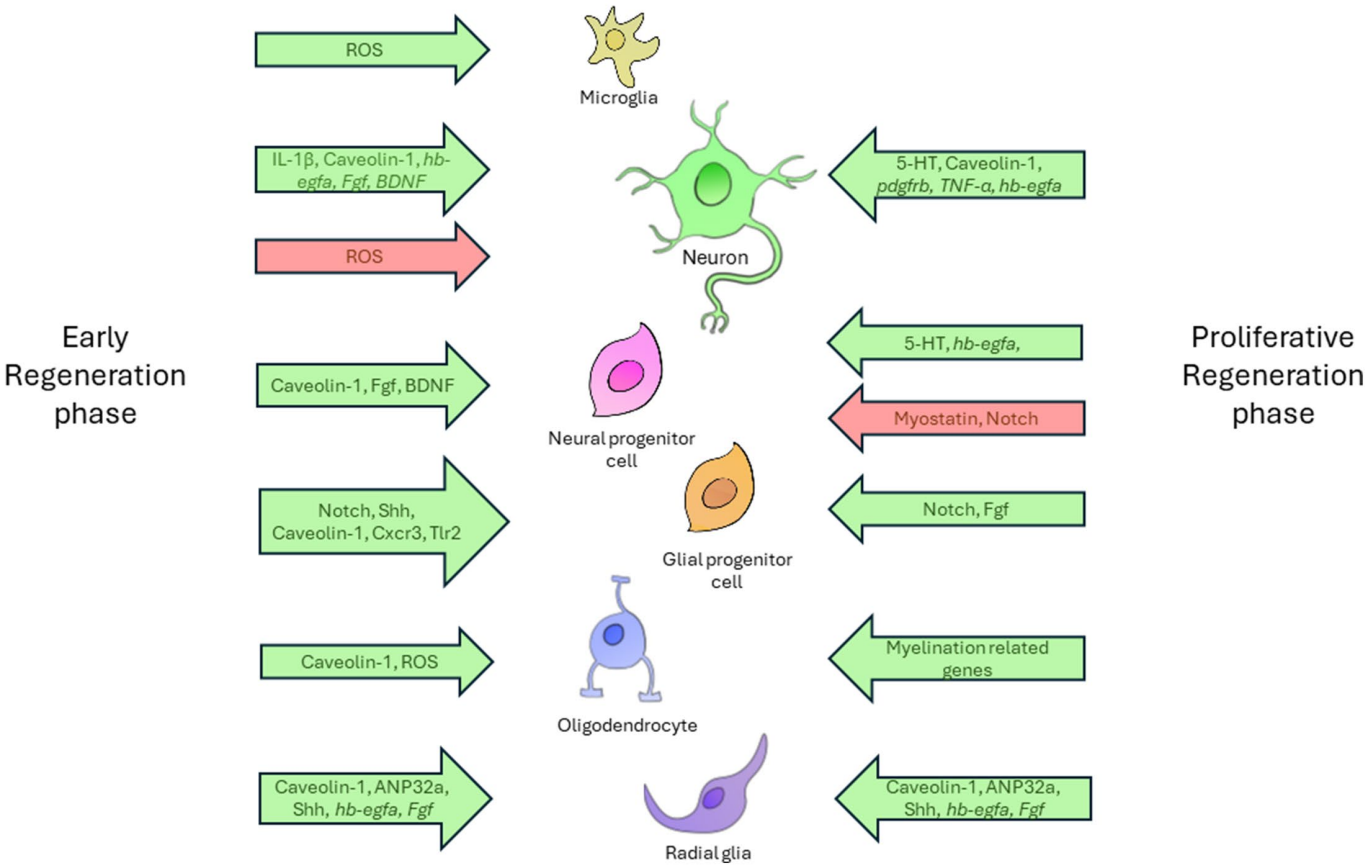
The diagram demonstrates complex relationships between diverse cell types and the regulatory factors involved in the nervous system regeneration in zebrafish. The regenerative process is provisionally divided into two distinct phases: the early phase and the proliferative phase. Each phase is characterized by specific cellular activities. The diagram shows the principal cell types involved in regeneration, including microglia, neurons, neuronal and glial progenitor cells, radial glia, and oligodendrocytes. The relationships between these cells and the influencing factors are illustrated using arrows that indicate the direction and nature of the factors’ effects. Permissive factors, which facilitate cellular processes, are designated by green arrows. These factors are instrumental in driving essential regenerative actions, including cell activation, axon growth, differentiation, and proliferation. In contrast, inhibitory factors, which impede these processes, are designated by red arrows. Factors located on the left of the timeline are associated with the early phase of regeneration and exert their effect at the initial stages of the cellular response. Those situated on the right correspond to the proliferative phase, where they regulate the later stages of regeneration, including cellular growth and differentiation.

As shown in one of the studies, the expression profiles of various signaling pathways associated with cell death, developmental and proliferation signals, inflammatory response, neuronal differentiation, and axon guidance undergo dynamic changes at different stages following spinal cord injury in zebrafish (Hui et al., 2014). Figure 2 shows a schematic representation of the signaling pathways and cell populations involved in neuroregeneration in zebrafish.

According to numerous studies, signaling pathways associated with the initial development of the organism are activated in response to damage. Therefore, the Wnt/*β*-catenin signaling plays a crucial role in early stages of regeneration, when it is directly linked to p53, MAPK, Fox-O, mTor, and apoptosis (Briona et al., 2015; Demirci et al., 2020), and in a later proliferative phase (Strand et al., 2016). The Wnt signaling pathway has a significant effect on the proliferation and differentiation of radial glia at the injury site, both in the early post-traumatic phase and in the proliferative phase of CNS regeneration (Shimizu et al., 2018). Wnt also influences axonogenesis by modifying the composition of extracellular matrix (Wehner et al., 2017). It is also worth noting that Wnt/β-catenin plays a crucial role in the process of retinal regeneration following traumatic injury in zebrafish (Meyers et al., 2012).

The Shh signaling pathway is critical for regeneration and important for both local and systemic neural damage. After stab wound injury in adult fish, the upregulation of Shh activates the proliferation of radial glia cells in the optic tectum. However, this process inhibits their differentiation into neurons (Ueda et al., 2018). Shh is activated in the cerebellum of adult zebrafish after blunt forced injury within the first few hours post-injury. However, by 60 hpi, it returns to the pre-injury expression level (Hentig et al., 2021b). After spinal cord injury in adult fish, Shh is significantly higher expressed in ependymo-radial glia in the ventral part of the spinal canal compared to control animals. This expression changes dynamically over time (Reimer et al., 2009). Shh activation has an anti-edematous and anti-inflammatory effect and reduces seizure activity in adult zebrafish with systemic traumatic brain injury (Hentig et al., 2021a).

*Notch* signaling, when overexpressed, influences the post-traumatic neurogenesis in the adult fish spinal cord by significantly reducing the number of new neurons (Dias et al., 2012). Histone deacetylases 1 and 3, in synergy with Notch, reduce the proliferative activity of radial glia and increase the post-traumatic neurogenesis in the optic tectum. This is achieved by affecting the expression of *her4.1* and *her6* (Kiyooka et al., 2020).

Various members of the FGF family influence the neurogenesis and axonogenesis in the zebrafish nervous system following experimental injury. Studies have demonstrated that FGF2, FGF3, and FGF8 are involved in the stimulation of neuronal progenitor cell proliferation, neurite outgrowth, and glial cell morphogenesis, thereby promoting axon repair after injury (Goldshmit et al., 2018). The interaction between the Wnt/β-catenin and FGF signaling pathways enhances proliferation and differentiation during neuromast regeneration in zebrafish, promoting the production of hair cells and supporting cells after injury (Tang et al., 2019). Overall, FGF signaling acts as a key player in the organization of neurogenesis and axon repair processes in zebrafish, demonstrating its importance in stimulating efficient neurogenesis and axon growth.

#### Other factors

Post-traumatic inflammation plays a crucial role in the post-transection spinal cord regeneration in both larval (Keatinge et al., 2021) and adult zebrafish (Saraswathy et al., 2022). Of particular note is the activation of TGF-β signaling pathways such as those in dorsal SOX2 positive progenitors in adults (Saraswathy et al., 2022), which appears to be one of a key factor in the successful spinal cord regeneration in larvae (Keatinge et al., 2021).

The myostatin expression is detected in SOX2 progenitor cells between days 7 and 14 after spinal cord injury. This factor is crucial for restoration of the functional characteristics of the spinal cord, but it has no effect on the restoration of the axonal structure or the formation of the glial bridge between the wound edges. Furthermore, it has been found to reduce the differentiation of progenitor cells into neurons in the dorsal part of the injury zone (Saraswathy et al., 2022).

In the process of regeneration (6–11 dpi), a significant increase in contactin-2 expression is observed following spinal cord transection in adult zebrafish. This has been found to be positively correlated with axon regrowth and functional recovery in injured animals. The same correlation is recorded from axons of nMLF neurons. Therefore, one can assume that proteins of the contact in family may exert a significant effect on the recovery of neural tissue post-injury (Lin et al., 2012).

Additionally, it has been demonstrated that the brain-derived neurotrophic factor (BDNF) expression in the telencephalon of adult zebrafish markedly increases at the 1-day mark following injury, which is then followed by a gradual decline at the 15-day mark. It is noteworthy that the expression of this protein differs from that observed after injury in rats. In zebrafish, it is significantly expressed in the injured hemisphere, whereas in mammals, it is expressed in the contralateral hemisphere. The authors associate this observation with the formation of glial scars in rats (Cacialli et al., 2018). The role of BDNF in the continuous regeneration and turnover of structures such as the “rosette” in the retina and taste buds of juvenile and adult teleosts has been demonstrated, and its importance in maintaining the functionality of sensory organs emphasized (Aragona et al., 2022). The above findings suggest that this factor may influence not only constitutive but also regenerative neurogenesis in different parts of the nervous system and may be a potential therapeutic agent to facilitate neuroregeneration.

The “bystander effect” in neurotrauma refers to the influence of injured neurons on neighboring, uninjured cells. In adult zebrafish, this effect assumes a pivotal significance in the context of spinal cord injury (SCI) and various forms of neural damage.

Calcium imaging techniques have revealed an elevation in resting calcium levels within motoneurons (pMNs) post-injury, indicating a disruption in calcium homeostasis during the regenerative process. This alteration is further correlated with a pronounced upregulation of Calretinin expression, thereby implying an essential role for this protein in the buffering of intracellular calcium concentrations.

Furthermore, the expression levels of connexin 35/36 have been observed to increase post-injury, indicating a potential enhancement in gap junction-mediated intercellular communication among neurons. These findings underscore the fundamental importance of intercellular and biochemical signaling processes in the preservation of neuronal integrity and facilitation of recovery. Subsequent investigations should explore in greater depth the molecular pathways implicated in the bystander effect to more thoroughly elucidate the mechanisms underlying neuroprotection and regeneration (Pedroni et al., 2024).

## Conclusion

The zebrafish (*D. rerio*) represents a valuable vertebrate model to study the nervous system. This organism has a number of advantages due to its morphological resemblance with mammals and regenerative abilities, which makes it a suitable choice for investigating a variety of traumatic injuries to the nervous system. Furthermore, both zebrafish larvae and adults can be used in the neuronal damage and neurogenesis research, providing a lot of opportunities for experimentation. In addition, this animal model offers a broad range of tools for disease modeling purposes. A variety of transgenic animal strains are currently available, and even double transgenic lines can be obtained to facilitate studies.

Like other model organisms, zebrafish has some limitations: its small size restricts applicability of such technologies as fMRI and EEG. Furthermore, zebrafish cannot perform cognitive tasks such as those set up for more complex animals. Nevertheless, the use of this fish in research markedly reduces costs and accelerates data acquisition. The regenerative abilities of zebrafish facilitate investigation of the fundamental mechanisms underlying the nervous system regeneration and potentially provide a basis for the development of therapies to treat injuries in various regions of the mammalian brain and spinal cord.

Zebrafish research has provided significant insights into neural regeneration, with, however, many issues to be addressed, which suggests the necessity of further studies. Despite the identification of the major signaling pathways involved in the nervous system regeneration in zebrafish, questions still remain as to their interactions during various stages of post-traumatic regeneration, in particular in aged individuals. Epigenetic modifications may also be of substantial significance, with DNA methylation patterns and non-coding RNAs exhibiting considerable variation between zebrafish (including various age and sex groups) and species with lower regenerative capacities.

While the responses of sensory and motor neurons to injury models have been extensively investigated, there have been a limited number of studies focused on the regeneration of interneurons and the re-establishment of the normal architecture of intricate neuronal networks. This should be subject to further investigation.

Environmental factors may also exert considerable effect on the regenerative capacities of organisms. A promising domain of investigation is the effects of these factors on the rate and completeness of regeneration. Furthermore, only very few studies have conducted long-term observations of animals to evaluate the morphological and functional alterations of cells within the regeneration zone and associated regions of the nervous system, which may be regarded as a promising subject for future research.
