# Supplementary material for: Modeling traumatic brain and neural injuries: insights from zebrafish
Source: Front Mol Neurosci. 2025 Mar 27;18:1552885. doi: 10.3389/fnmol.2025.1552885 (PMC11983547; doi:10.3389/fnmol.2025.1552885)
Supplement: Supplementary file 1 [file Table_1.DOCX]

Table 1: Overview of Techniques for Modeling Neural System Trauma in Zebrafish Across Developmental Stages

| **Method** | **Age** | **Tool** | **Location** | **Author** |
| --- | --- | --- | --- | --- |
| Traumatic brain injury modeling | adult | Marmarou weight drop | whole brain | (Maheras et al., 2018), (Hentig et al., 2021b) |
| Closed-head model of TBI | adult | pulsed high intensity focused ultrasound (pHIFU) | whole brain | (McCutcheon et al., 2017) |
| Closed-head model of TBI | larva | syringe with a closed valve stopper | whole nervous system | (Alyenbaawi et al., 2021) |
| Closed-head model of TBI | larva | linear acceleration–deceleration motion | whole nervous system | (Beppi et al., 2022) |
| Stab wound | adult | syringe needle (26 3/8-gauge, 0.2 mm) | hindbrain | (Hannah et al., 2012) |
| Stab wound | adult | sterile 27-gauge needle | telencephalon | (Kishimoto et al., 2012), (Kim et al., 2020), (Lübke et al., 2022) |
| Stab wound | adult, larva | sterile 27–31-gauge needle or glass capillary | optic tectum | (Gan et al., 2020), (Ueda et al., 2018), (Shimizu et al., 2018) |
| Stab wound | adult | sterile 27-gauge needle | cerebellum | (Wu et al., 2014) |
| Laser ablation | adult | laser diode | telencephalon | (Tikhonova et al., 2022) |
| Transection | adult | scissors | spinal cord | (Becker et al., 1998), (Vajn et al., 2014), (Fang et al., 2012), (Hui et al., 2014) |
| Crush | adult | forceps | spinal cord | (Hui and Ghosh, 2016) |
| Crush | adult | forceps | optic nerve | (Diekmann et al., 2015), (Meehan et al., 2023) |
| Transection | adult, aged | glass capillary | posterior lateral line | (Graciarena et al., 2014) |
| Electroablation | adult, larva | microelectrode | axon | (Moya-Díaz et al., 2014) |
| Transection | larva | glass capillary | spinal cord | (Briona and Dorsky, 2014) |
| Transection and puncture | larva | needle | spinal cord | (Ohnmacht et al., 2016), (John et al., 2022), (Hossainian et al., 2022) |
| Transection | larva | needle | optic nerve | (Harvey et al., 2019), (Harvey et al., 2023) |
| Laser ablation | larva | two-photon excitation laser | fluorescent labeled cells of nervous system | (Liu and Fetcho, 1999), (Roeser and Baier, 2003),  (Graciarena et al., 2014), (Muto and Kawakami, 2018), (Hiyoshi et al., 2021), (Isabella et al., 2021), (Xiao et al., 2022) |

Table 1 summarizes different techniques used to model a neural system trauma in zebrafish of various ages. It is important to note that the whole-brain trauma model is more frequently used for adult zebrafish and less frequently for larvae. Stab wounds are selected to induce open-head trauma in specific brain areas. Linear structures such as the spinal cord, lateral line, and optic nerve can be injured using different tools in animals of various ages. Microinvasive interventions, resulting in single-cell injury, can be carried out using two-photon excitation lasers and microelectrodes.
